# Redo renal denervation using a multi-electrode radiofrequency system in patients with persistent therapy-resistant hypertension

**DOI:** 10.1007/s12471-017-0986-z

**Published:** 2017-04-12

**Authors:** J. Daemen, L. Feyz, L. Van Zandvoort, N. M. Van Mieghem

**Affiliations:** Department of Interventional Cardiology, Erasmus Medical Center, Rotterdam, The Netherlands

**Keywords:** Sympathetic denervation, Hypertension, Catheter ablation

## Abstract

**Objectives:**

Renal sympathetic denervation has been studied as a potential therapeutic option for patients with therapy-resistant hypertension; however, a significant proportion of patients do not show a significant reduction in blood pressure and are classified as non-responders. The objective of the present study was to assess whether a redo renal denervation procedure increases response rates.

**Methods:**

We present a case series of three consecutive renal denervation non-responders treated with the multi-electrode radiofrequency St. Jude EnligHTN catheter after an average of 22 months. Patients were followed for 6 months.

**Results:**

Mean age was 66 years and two patients were male. Patients were previously treated using either ReCor’s Paradise system, the Vessix V2 system or the Covidien OneShot system. Mean office blood pressure one year after the initial procedure was 187/102 mm Hg with a mean 24 h ambulatory blood pressure of 166/102 mm Hg. All patients underwent a successful redo procedure using the EnligHTN system because of persistent therapy-resistant hypertension. At 6 months a significant drop in both office and ambulatory blood pressure of −27/−6 mm Hg and −15/−13 mm Hg, respectively, was observed. No significant renal artery stenosis was observed at 6 months.

**Conclusions:**

In patients with therapy-resistant hypertension who do not respond to an initial renal denervation procedure, a redo procedure using the St. Jude EnligHTN system may help to significantly improve blood pressure control.

## Introduction

Controlling blood pressure in hypertensive patients remains a challenge and treatment targets are frequently not achieved despite multiple antihypertensive drugs [1]. Renal sympathetic denervation (RDN) has been studied as a potential therapy to decrease blood pressure in patients with therapy-resistant hypertension [2–4]. Several catheter-based RDN techniques exist and proof of adequate denervation and efficacy is mounting [5, 6]. However, the individual magnitude of the treatment effect varies extensively, with a significant proportion of patients not responding to the therapy in terms of blood pressure reduction – irrespective of the technology used. A substantial number of both patient and procedural characteristics have been hypothesised to account for this so-called non-responsiveness [7, 8]. While characteristics such as older age and a non-compliant arterial system as well as inadequate renal nerve destruction on treatment with first generation devices are clear, theoretical causes of this non-responsiveness, scientific evidence is still scarce. Previous work has already demonstrated that a higher degree of renal nerve disruption leads to greater inhibition of the sympathetic nerve system [9]. Building further on this hypothesis, performing a redo procedure could theoretically make sense in a proportion of the patients who do not respond to the initial RDN procedure. Apart from one case report on a successful redo procedure using the Symplicity Flex system, and one study on the effect of cryoablation as a second line option in RDN non-responders, no data on the additional effect of a redo RDN procedure on blood pressure reduction are available [10, 11]. We present a series of three consecutive RDN non-responders who underwent a second procedure using the next generation multi-electrode St. Jude Medical EnligHTN system.

## Methods

### Patient selection and definitions

Three consecutive patients who did not show a relevant reduction in both office and ambulatory blood pressure after renal sympathetic denervation because of therapy-resistant hypertension are presented. All procedures were carried out at the Erasmus Medical Center, Rotterdam, the Netherlands, between December 2012 and March 2013. Initial denervation was performed with the Paradise system (ReCor Medical, Palo Alto, CA), the OneShot system (Covidien, Campbell, CA) and the Vessix V2 system (Boston Scientific, Natick, MA), respectively [12]. In order to qualify as non-responders, patients had to fulfil the following criteria: mean systolic 24 h ambulatory blood pressure >150 mm Hg despite treatment with >3 antihypertensive drugs or failure to show a reduction of >10 mm Hg in mean systolic 24 h ambulatory blood pressure at 12 months after the initial RDN procedure.

Office blood pressure recordings were collected in accordance with the Standard Joint National Committee VII Guidelines and ESC/ESH Guidelines [13]. Twenty-four hour ambulatory blood pressure was obtained using an Ambulatory Blood Pressure System (Spacelabs Healthcare, Inc., Issaquah, WA, USA).

Redo procedures were performed at 22.3 ± 4.6 months after the initial procedure. At the time of the redo procedure all patients were free of adverse events related to the index procedure, showed no signs of renal artery stenosis – diagnosed using either computed tomography (CT) or magnetic resonance (MR) angiography – and had preserved renal function.

#### Redo denervation procedure

All patients were preloaded with 300 mg aspirin, if naïve, and advised to continue with aspirin for at least 1 month. All procedures were performed under conscious sedation using 1 to 3 mg of midazolam and 50 to 100 µg of fentanyl. After local anaesthesia, common femoral artery access was obtained by echo-guided puncture and a 6 Fr sheath was introduced. Under fluoroscopic guidance the short 6 Fr sheath was exchanged for an 8 Fr RDN or IMA tipped guiding sheath, which is recommended for the easier use of the St. Jude EnligHTN system. Pre-procedurally, 100 IU heparin/kg were administered to achieve an active clotting time >250 s. After engaging the renal arteries by using a no-touch technique with the help of a standard high-torque Balance Heavyweight (BHW) coronary guidewire, selective renal artery angiograms were made and an appropriate basket size was chosen (small basket 4.0–5.5 mm diameter/large basket 5.5–8.0 mm diameter). The BHW guidewire was exchanged for the EnligHTN ablation catheter and its tip positioned proximally to the bifurcation of the main renal artery. The basket catheter, containing four bipolar platinum-iridium electrodes, was then opened and the impedance of each electrode on the basket was monitored. After a total of four ablations were performed successfully the basket was collapsed and retracted proximally while another four ablations were performed in the same artery. A total of eight ablations were performed in each vessel, except for one artery in one patient in which the basket could not be engaged due to the very steep take-off of the right renal artery.

#### Follow-up

All patients were discharged home the following day. All patients were followed according to a dedicated follow-up protocol including office visits at 1, 3 and 6 months, office blood pressure measurement at each time point and ambulatory blood measurements at 3 and 6 months. Additionally, a 12-lead ECG, blood and urine collections and a 6-month assessment of renal artery patency using either CT or MR angiography were done in all patients.

## Results

Patient demographics and baseline characteristics are depicted in Table 1. Procedural characteristics in Table 2 and clinical follow-up parameters in Table 3.

**Table 1.** Baseline characteristics

	Patient 1	Patient 2	Patient 3
Age	76	59	60
Gender	Female	Male	Male
Race	Hindu	Caucasian	Caucasian
Length, cm	168	196	170
Weight, kg	99	98	76.9
BMI, kg/m^2^	35.2	25.6	26.6
Mean office blood pressure (mm Hg)	193/93	173/115	195/98
Mean ambulatory blood pressure (mm Hg)	173/101	161/107	164/98
Diabetes	No	No	Yes
Hypercholesterolaemia	No	No	Yes
Current smoking status	No	No	Yes, 20 PY
eGFR, ml/min	56	72	90
Coronary artery disease	No	No	No
Peripheral artery disease	Yes	No	No
History of stroke	Yes	No	No
*Antihypertensive medication*			
Ace inhibitor	Yes	No	No
Angiotensin receptor blocker	No	Yes	Yes
Diuretic	Yes	Yes	Yes
Calcium channel blocker	Intolerant	Yes	Yes
Alpha blocker	No	Yes	Yes
Beta blocker	Yes	Yes	Yes
Aldosterone receptor antagonist	No	No	No
Vasodilator	No	No	Yes
Previously used RDN device	Recor Paradise 3 ablations/artery	Vessix 8 ablations/artery	Covidien 1 ablation/artery
Office blood pressure before initial treatment	207/93	165/100	184/103
Ambulatory blood pressure before initial treatment	155/75	161/109	162/98
Change in office blood pressure 1 year after 1^st^ procedure	−14/0	+8/+15	+11/−5
Change in ambulatory blood pressure after 1^st^ procedure	+18/26	0/−2	+2/0

**Table 2.** Redo procedure characteristics

	Patient 1	Patient 2	Patient 3
Number of ablations	8 (left side only)	8 + 8	8 + 8
Duration of the procedure, min	64	81	45
Contrast usage, ml	70	120	90
Renal artery dissection	No	No	No
Renal artery spasm	No	No	Yes
Renal artery thrombus	No	No	No

**Table 3.** Clinical follow-up parameters

	Patient 1		Patient 2		Patient 3	
	3 months	6 months	3 months	6 months	3 months	6 months
Drop in mean office blood pressure (mm Hg)	−31/+2	−62/−18	+8/−5	+28/+10	−50/−23	−30/−10
Drop in ambulatory blood pressure (mm Hg)	−44/−28	−16/−22	−2/+3	−9/−4	−2/−4	−20/−12
Change in heart rate (bpm)	0	0	−8	−2	−19	−9
Change in eGFR (ml/min)	0	+5	+6	−5	0	0
*Antihypertensive medication*						
Ace inhibitor	Yes	Yes	No	No	No	No
Angiotensin receptor blocker	No	No	Yes	Yes	Yes	Yes
Diuretic	Yes, dose decreased	Yes	Yes	Yes, dose increased	No	No
Calcium channel blocker	Intolerant	Intolerant	Yes	Yes	No	No
Alpha blocker	No	No	Yes	Yes	No	No
Beta blocker	Yes	Yes	Yes	Yes	Yes	Yes
Aldosterone receptor antagonist	No	Yes	No	No	No	No
Vasodilator	No	No	No	No	No	No
*Adverse events*						
Death	No	No	No	No	No	No
Myocardial infarction	No	No	No	No	No	No
Stroke/TIA	No	No	No	No	No	No

Patient 1 was a 76-year-old obese female with persistent headaches, known atrial fibrillation and hypertension since the age of 36. Due to repeated episodes of life-threatening gastrointestinal bleeding coumadin was stopped and replaced by aspirin. Secondary causes of hypertension were excluded. With a mean office blood pressure of 207/93 mm Hg (24 h ambulatory blood pressure monitoring 155/75 mm Hg) despite the use of three antihypertensive drugs, she underwent a first RDN using the ReCor Paradise system. After an uneventful recovery her blood pressure remained uncontrolled and she suffered from an ischaemic stroke. One year after the initial treatment, office blood pressures remained 193/93 mm Hg and given her clearly hypertension-related comorbidities, a second procedure combining a redo RDN procedure and a percutaneous left atrial appendage closure was performed. Due to the very steep take-off of her right renal artery only a left-sided ablation could be performed successfully. After a successful recovery, office blood pressures significantly dropped to 131/75 mm Hg (ambulatory 129/73 at 3 months, 157/79 mm Hg at 6 months and 110/64 mm Hg at 12 months). The headaches resolved and renal function increased from 56 ml/min at baseline to 61 ml/min at 6 months.

Patient 2 was a 59-year-old Caucasian male with persistent headaches and a 40-year history of hypertension for which he received six antihypertensive drugs. Renal function was preserved. With a mean office blood pressure of 165/100 mm Hg and a 24 h ambulatory blood pressure of 161/109 mm Hg he underwent a successful bilateral RDN procedure using the Vessix V2 RDN system after secondary causes of hypertension were excluded. One year after the initial procedure his clinical condition remained stable with no improvement in blood pressure control. Both office and ambulatory blood pressure remained unchanged (office mean 173/115 mm Hg, ambulatory 161/107 mm Hg) despite a stable medication regimen. Given his persistent headaches and persistent refractory hypertension, a redo denervation was scheduled. After intensive follow-up at 1, 3 and 6 months, ambulatory blood pressures started to decrease slightly to 159/110 at 3 months and 152/103 at 6 months, while office blood pressure remained stable.

Patient 3 was a 60-year-old Caucasian male with a history of insulin-dependent diabetes, hypercholesterolaemia and hypertension of more than 10 years. The patient was a heavy smoker (over 20 pack-years). Despite two percutaneous coronary interventions, the patient suffered from stable angina CCS Class II and severe fatigue. Both renal and cardiac functions were preserved. The patient was on six antihypertensive drugs and was referred for RDN with an office and ambulatory blood pressure of 184/103 mm Hg and 162/98 mm Hg, respectively. One year after a successful bilateral RDN using the Covidien OneShot system, blood pressures remained unchanged and angina worsened to class III. Given his extensive number of comorbidities, persistent grade III hypertension with a mean office blood pressure of 195/98 mm Hg (ambulatory 164/98 mm Hg), a redo procedure was scheduled in combination with a further coronary angiogram. After a successful bilateral RDN using the EnligHTN system, the coronary angiogram showed severe three-vessel disease. The patient was discussed by the heart team and accepted for coronary artery bypass surgery which was performed soon thereafter. The patient recovered quickly and uneventfully. Along with a significant reduction in both office and ambulatory blood pressure to 165/99 mm Hg and 146/86 mm Hg his symptoms of fatigue resolved and angina reduced to class I. The number of antihypertensive drugs was reduced to two at 6 months.

## Discussion

In the present case series, we report the outcome of three consecutive patients with persistent grade III hypertension despite an initial technically successful RDN procedure one year previously, while still being on optimal medical therapy. A second RDN procedure using the St. Jude EnligHTN system proved to be safe in all patients with no procedure-related adverse events up to 6 months. Drops in ambulatory systolic blood pressure ranged from 9 mm Hg in patient 2, to 20 mm Hg in patient 3.

Uncontrolled hypertension is independently associated with the incidence of stroke, coronary and peripheral artery disease, heart failure, sudden death and renal insufficiency [14, 15]. Conversely, only a small proportion of hypertensive patients have elevated blood pressure alone; the majority also suffer from additional cardiovascular risk factors. Furthermore, the presence of hypertension and other cardiovascular risk factors may potentiate one another, resulting in an exponentially elevated risk for future adverse events [13]. The latter is reflected by the patients presented in this case series, in which persistently uncontrolled blood pressure might have contributed to their comorbidities and adverse events. Despite extensive follow-up at a tertiary referral centre, where secondary causes of hypertension were excluded in all patients and drug regimens were improved, blood pressure levels remained unacceptably high. This illustrates the need for more effective antihypertensive therapies in patients with drug-resistant hypertension. Over the past years, a wide variety of invasive and non-invasive treatment options with the intention to reduce sympathetic nervous system activity and thereby improve blood pressure control, have been evaluated [16, 17]. Of these, percutaneous RDN is currently the most widely studied option with studies reporting 24 h ambulatory systolic blood pressure reduction in the range of 2 to 16 mm Hg [4, 18, 19]. However, individual treatment response varied extensively from unchanged hypertension and thus non-responsiveness, to frank hypotension and the need to taper pharmacological antihypertensive treatment. The frequency of non-responder rates reportedly varies between 15 and 30% where the definition of a lack in decrease in office systolic blood pressure >10 mm Hg was used [9, 20, 21].

Despite the growing body of evidence supporting the potential additional value of renal sympathetic denervation in patients with therapy-resistant hypertension, responders have not been clearly identified yet. The hypothesis of the present study was to test if a second denervation using the multi-electrode EnligHTN system could lead to a higher degree of sympathetic nerve damage and subsequently lead to a higher level of blood pressure reduction as a surrogate endpoint. Two previous studies have evaluated the effectiveness of a second denervation procedure. A case report by Lambert et al. reported on a 79-year-old hypertensive patient who experienced a drop of 40 mm Hg in systolic office-based blood systolic blood pressure 6 months after an initial procedure using the Medtronic Symplicity system [11]. With the blood pressure back to baseline levels at 1‑year after the procedure the decision was made to perform a redo procedure using the same device. Three months later, office systolic blood pressure levels were again 40 mm Hg lower. This case suggests that the redo denervation procedure had a positive effect, but also illustrates the high variability of office blood pressure thus precluding any firm conclusions. A second study evaluated the safety and effectiveness of cryoablation in ten RDN non-responders [10]. The study reported an impressive reduction in both mean systolic office blood pressures as well as ambulatory blood pressure of 61 mm Hg and 52 mm Hg, respectively, at 12 months.

In the Symplicity HTN-I study, a mean reduction in renal norepinephrine spillover of 47% was reported. However, the 95% confidence interval ranged from 28 to 65% [20]. The hypothesis that this first generation device was potentially not as technically efficacious as initially hypothesised was soon confirmed by interesting post-mortem work by Vink et al. [22]. In a patient who died 12 days after an RDN procedure, the authors demonstrated a dome-shaped distribution field with limited penetration leaving a large part of the nerves in periadventitial areas unaffected. The authors concluded that it was unlikely that RDN using the first generation Symplicity device would result in complete interruption of the continuity of all adventitial nerve bundles around the renal arteries. As a consequence, several new devices quickly appeared on the market with data showing substantially higher degrees of periadventitial nerve damage along with higher drops in norepinephrine levels [5, 6]. Remarkably, non-responder rates did not clearly improve, suggesting that specific patient-related factors might also account for part of the non-response. The fact that the redo procedure was effective in all three patients in the present study creates a new dilemma since all of them were treated with second-generation devices. On the other hand, it suggests that a redo procedure could be an option for all non-responders – at least for now.

To the best of our knowledge this is the first report of the potential performance of a redo RDN procedure using the EnligHTN RDN system in three patients with persistent refractory hypertension. Despite their rather distinct baseline characteristics and risk profile, the redo procedure significantly helped in improving blood pressure control and physical condition in two patients and modestly reduced blood pressure in one patient. Given the lack of validated alternative treatment options in these high-risk patients, we believe that a redo RDN using the EnligHTN system can be a safe and effective option to improve blood pressure and potentially reduce future adverse cardiovascular events.

### Limitations

The present study was limited by the small number of patients with extensive comorbidities. Two of the three patients received additional treatment in combination with the redo denervation procedure. However, it is unlikely that the left atrial appendage closure and/or coronary artery bypass surgery led to the significant blood pressure reductions observed in both patients with a 20-year history of hypertension. Finally, we cannot confirm that the redo procedure might have had a similar effect if devices other than the EnligHTN system had been used.

## Conclusion

Redo RDN using the EnligHTN system may be a safe and effective option to improve blood pressure control in patients with persistent therapy-resistant hypertension after the initial RDN procedure.
